# Behavioural economic interventions to reduce health care appointment non-attendance: a systematic review and meta-analysis

**DOI:** 10.1186/s12913-023-10059-9

**Published:** 2023-10-23

**Authors:** Kalin Werner, Sara Abdulrahman Alsuhaibani, Reem F. Alsukait, Reem Alshehri, Christopher H. Herbst, Mohammed Alhajji, Tracy Kuo Lin

**Affiliations:** 1 Institute for Health & Aging, Department of Social and Behavioral Sciences, University of California, San Francisco, San Francisco, CA USA; 2 Nudge Unit, Ministry of Health, Riyadh, KSA Saudi Arabia; 3 Department of Health Sciences, College of Health and Rehabilitation Sciences, Princess Nourah bint Abdulrahman University, Riyadh, KSA Saudi Arabia; 4 Community Health Sciences, College of Applied Medical Sciences, King Saud University, Riyadh, KSA Saudi Arabia; 5 Health, Nutrition and Population Global Practice, The World Bank, Washington, D.C USA; 6 College of Medicine, Alfaisal University, Riyadh, KSA Saudi Arabia

**Keywords:** Behavioural economics, No-show rates, Reminders, Attendance rates, Systematic reviews

## Abstract

**Background:**

Appointment non-attendance – often referred to as “missed appointments”, “patient no-show”, or “did not attend (DNA)” – causes volatility in health systems around the world. Of the different approaches that can be adopted to reduce patient non-attendance, behavioural economics-oriented mechanisms (i.e., psychological, cognitive, emotional, and social factors that may impact individual decisions) are reasoned to be better suited in such contexts – where the need is to persuade, nudge, and/ or incentivize patients to honour their scheduled appointment. The aim of this systematic literature review is to identify and summarize the published evidence on the use and effectiveness of behavioural economic interventions to reduce no-shows for health care appointments.

**Methods:**

We systematically searched four databases (PubMed/Medline, Embase, Scopus, and Web of Science) for published and grey literature on behavioural economic strategies to reduce no-shows for health care appointments. Eligible studies met four criteria for inclusion; they were (1) available in English, Spanish, or French, (2) assessed behavioural economics interventions, (3) objectively measured a behavioural outcome (as opposed to attitudes or preferences), and (4) used a randomized and controlled or quasi-experimental study design.

**Results:**

Our initial search of the five databases identified 1,225 articles. After screening studies for inclusion criteria and assessing risk of bias, 61 studies were included in our final analysis. Data was extracted using a predefined 19-item extraction matrix. All studies assessed ambulatory or outpatient care services, although a variety of hospital departments or appointment types. The most common behaviour change intervention assessed was the use of reminders (n = 56). Results were mixed regarding the most effective methods of delivering reminders. There is significant evidence supporting the effectiveness of reminders (either by SMS, telephone, or mail) across various settings. However, there is a lack of evidence regarding alternative interventions and efforts to address other heuristics, leaving a majority of behavioural economic approaches unused and unassessed.

**Conclusion:**

The studies in our review reflect a lack of diversity in intervention approaches but point to the effectiveness of reminder systems in reducing no-show rates across a variety of medical departments. We recommend future studies to test alternative behavioural economic interventions that have not been used, tested, and/or published before.

**Supplementary Information:**

The online version contains supplementary material available at 10.1186/s12913-023-10059-9.

## Introduction

Appointment non-attendance – often referred to as “missed appointment”, “patient no-show”, or “did not attend (DNA)” – causes volatility in health systems around the world. Empty and unfilled timeslots, which could otherwise be used if patients were to show up for appointments as scheduled, lead to unnecessary staffing expenses and revenue losses. Failure to attend scheduled appointments may also contribute to inefficient use of limited health care resources, worsening patient access and healthcare quality. For example, the National Health Service (NHS) in the United Kingdom has reported that patients who miss their general practitioner appointments alone cost the NHS around £216 million a year [1]. The cost and concern regarding patient no-show is so tremendous that introducing a fine for NHS patient no show became a salient issue during the 2022 Conservative party leadership bid [2].

Addressing the issue of non-attendance is essential to improving access and safeguarding limited health care resources. Furthermore, it may serve to reduce disparity in healthcare amongst minorities and patients with major mental illness and medically complex care whom have an increased likelihood of missed appointments. [3] Many studies have identified predictors of non-attendance [4, 5], and many of these predictors characterize the issues patients are likely to experience, and as a consequence, are more likely to miss an appointment, because of their sociodemographic characteristics. As such, it is critical to focus on ways that may mitigate issues related to non-attendance.

The problem of non-attendance can be attributed to numerous reasons including physical barriers to access (e.g., lack of affordable transportation [6], absence of childcare [7]), opportunity cost (e.g., the time required to seek care), and patient forgetfulness [8]. Moreover, behavioural science indicates that often patients do not behave in the way we would expect, and that behavioural factors such as limited attention, cognitive overload, and avoidance can impede timely care seeking and influence motivation to honour appointments. For example, in some circumstances feelings of fatalism and fear of negative outcomes have been found to act as a barrier to patients attendance of health screenings [9]. By understanding the psychological, emotional, cognitive, social factors that may influence patients’ decisions, behavioural insights can be applied to health system planning and guide policy design around appointment attendance.

Behavioural economic informed interventions are defined as an intervention designed to change behaviour within a decision context by counteracting psychological and cognitive biases or leveraging them for better decision making [10, 11]. Individuals may satisfice and choose sub-optimal options that may be against their own best interest. Behavioural economic insights can contribute to developing policies which encourage or guide behaviour without limiting free choice [12, 13]. Of the different approaches to reduce patient non-attendance, it can be reasoned that behavioural economics insights are best suited to understand how choice problems are optimized or solved to motivate patients to attend their scheduled appointment. Strategies to circumvent these barriers could modify choice architecture by directly removing the physical barriers (e.g., provide free transportation) and making it easier for patients to attend their appointments. Concomitantly, interventions may leverage behavioural economics theory and mechanisms to encourage individuals towards the desired behaviour (i.e., honour appointments).

Ways to encourage, persuade, and/or nudge patients to honour their scheduled appointments include providing resources to circumvent barriers to access (e.g., arranging transportation to healthcare clinics), providing information by reminding patients about their appointment (e.g., text message reminders), and financial incentives (e.g., a gift card for attending scheduled appointment). There is some evidence to suggest that even subtle encouragement that minimizes attentional biases, such as modified Short Message Service (SMS) reminders with details of the cost of missed appointments, can have meaningful impacts on behaviour [14].

Existing systematic reviews have indicated that the range of interventions proposed to reduce non-attendance all have a modest effect, but fail to summarize the key behavioural mechanism that impacts patient decision-making [15, 16]. In particular, when examining the evidence on the expected effect of the use of economic incentives, defined by a material gain or loss, remains sparse and mixed [17, 18]. The aim of this systematic literature review is to focus on and summarize the published evidence on the use and effectiveness of behavioural economics-related interventions to reduce no-shows for health care appointments; these studies may focus on one behavioural economic interventions, combine an behavioural economic intervention with a none behavioural economic intervention, or utilize an intervention to change behaviour by leveraging rationale from behavioural economics. The findings will contribute to evidence-based policymaking regarding interventions to reduce non-attendance and inform the development of future interventions in the healthcare sector.

## Methods

### Study design

We conducted a review following Preferred Reporting Items for Systematic Reviews and Meta-Analyses (PRISMA) reporting standards and registered with PROSPERO (CRD42022320844) [19]. We systematically searched four databases (PubMed/Medline, Embase, Scopus, and Web of Science) for published and grey literature on behavioural economic strategies to reduce no-shows for health care appointments. The authors used a combination of MeSH and text word searches to develop search strings to cover the following concepts: (1) patients scheduling and appointments, (2) no-show patients, and (3) behavioural economics. The complete search strategy is available in Supplementary 1, and an example of terms used in our search is provided in Box 1.

**Table:** 

**Box 1. PubMed search strings**
Appointments and Schedules OR Appointments, Patient OR schedules, patient [MeSH] OR “schedules and appointments” OR “schedules” OR “schedule” OR “patient schedules” OR “patient schedule” OR “schedule, patient” OR “appointments” OR “appointment” OR “patient appointments” OR “patient appointment” OR “medical appointment” AND
No-show patients [MeSH] OR “no show” OR “no-show” OR “non-attendance” OR “missed appointment*” OR “fail to attend” OR “failed to attend” OR “cancell*” AND
Economics, Behavioral [MeSH] OR “financial incentive” OR “financial penalt*” OR “fine” OR “penalt*” OR “monetary sanction” OR “behavioural economics” OR “behavioral economics” OR “asymmetric paternalism” OR “nudg*” OR “choice architect*” OR “reframe” OR “loss aversion” OR “endowment” OR “prospect theory” OR “feedback” OR “social comparison” OR “social norm” OR “active choice” OR “prompted choice” OR “accountable justification” OR “suggested alternative” OR “mental accounting” OR “allocation bias” OR “reminders” OR “salience” OR “commit*” OR “precommitment”

### Eligibility criteria

Eligible studies met four criteria for inclusion; they were (1) available in English, Spanish, or French, (2) assessed behavioural economics interventions, (3) objectively measured a behavioural outcome (as opposed to attitudes or preferences), and (4) used a randomized and controlled or quasi-experimental study design to enable causal inference. Studies which were non-randomized controlled study designs needed to control for relevant patient and care setting characteristics to be considered in our review. Since patient non-attendance is not a novel issue, our search did not limit based on date, and we aimed to capture all potential literature on the topic. Conference abstracts, posters, or protocols were excluded from the review. Although the systematic reviews did not fall within our inclusion criteria, we searched the references lists of topical reviews for any additional relevant studies. Duplicate removal, voting consensus, and extraction was conducted using Covidence systematic review software (Veritas Health Innovation, Melbourne, Australia. Available at www.covidence.org). For relevance assessment, two of three reviewers used the inclusion criteria and independently assessed each study for relevance, first by title and abstract and subsequently by screening available full texts. We followed definitions of key terms in determining the eligibility of studies (Table 1). We did not consider switching care services from in-person to telehealth virtual care appointments, as behavioural economic interventions. However, we included studies which integrated the use of telehealth services, such as online appointment management services, to existing in-person appointment services as a means to reduce opportunity costs for patients. Reviewers checked all within-publication references to identify additional sources. As a desk-based review, no ethical approval was sought.

**Table 1:** Key definitions

Term	Definition
Behavioural economics informed intervention	An intervention designed to change behaviour within a decision context by counteracting psychological and cognitive biases or leveraging them for better decision making.
Patient no-show *or* did-not-attend	When a patient does not present for a scheduled appointment, and did not cancel ahead of time (when applicable) [4, 20].

### Risk of bias assessment

A single reviewer assessed each study for risk of bias using the Mixed Methods Appraisal Tool (MMAT) [21]. The tool uses seven criteria based on study design for items ranging from clear research question, appropriate randomization, representativeness of target population, and confounders being accounted for in design and analysis. Scores of this assessment can be found in Appendix 1. Studies that achieved lower than 70% (4 out of 7) of the MMAT list of the appropriate study design were considered at high risk of bias and poor quality and, therefore, excluded from our review.

### Data extraction

Studies that met our inclusion criteria were then divided amongst the team of three reviewers for data extraction, using a predefined 19-item extraction matrix. The following variables were extracted; the aim of the study, the study setting/country, study design, description of population and total n, targeted hospital department or appointment type, intervention, time frame and frequency of intervention, mechanism of intervention, financial or non-financial intervention, comparators, outcomes measured, results, effect-size and confidence interval (either reported as the odds ratio or co-efficient). Summary of the attributes of included studies is presented in Appendix 2. Two reviewers discussed the variables of each study extracted using the matrix for final consensus.

### Data analysis

Study outcomes were first summarised in a narrative synthesis. Where it was appropriate to combine studies, such as the use of SMS texts as reminders, meta-analyses were conducted.

Only studies for which reported odds ratios, 95% confidence intervals, and study population size were included in the meta-analysis. We used a random-effects model, assuming that study effect sizes differ, to estimate the mean distribution of affects. This approach allows for a more equal weighting than fixed-effects models [22]. Heterogeneity was examined using Cochran’s Q test and Higgin’s I2 test. Weighted effect sizes for each study were calculated using inverse-variance weight and plotted using Microsoft Excel (2022).

## Results

**Fig. 1:**
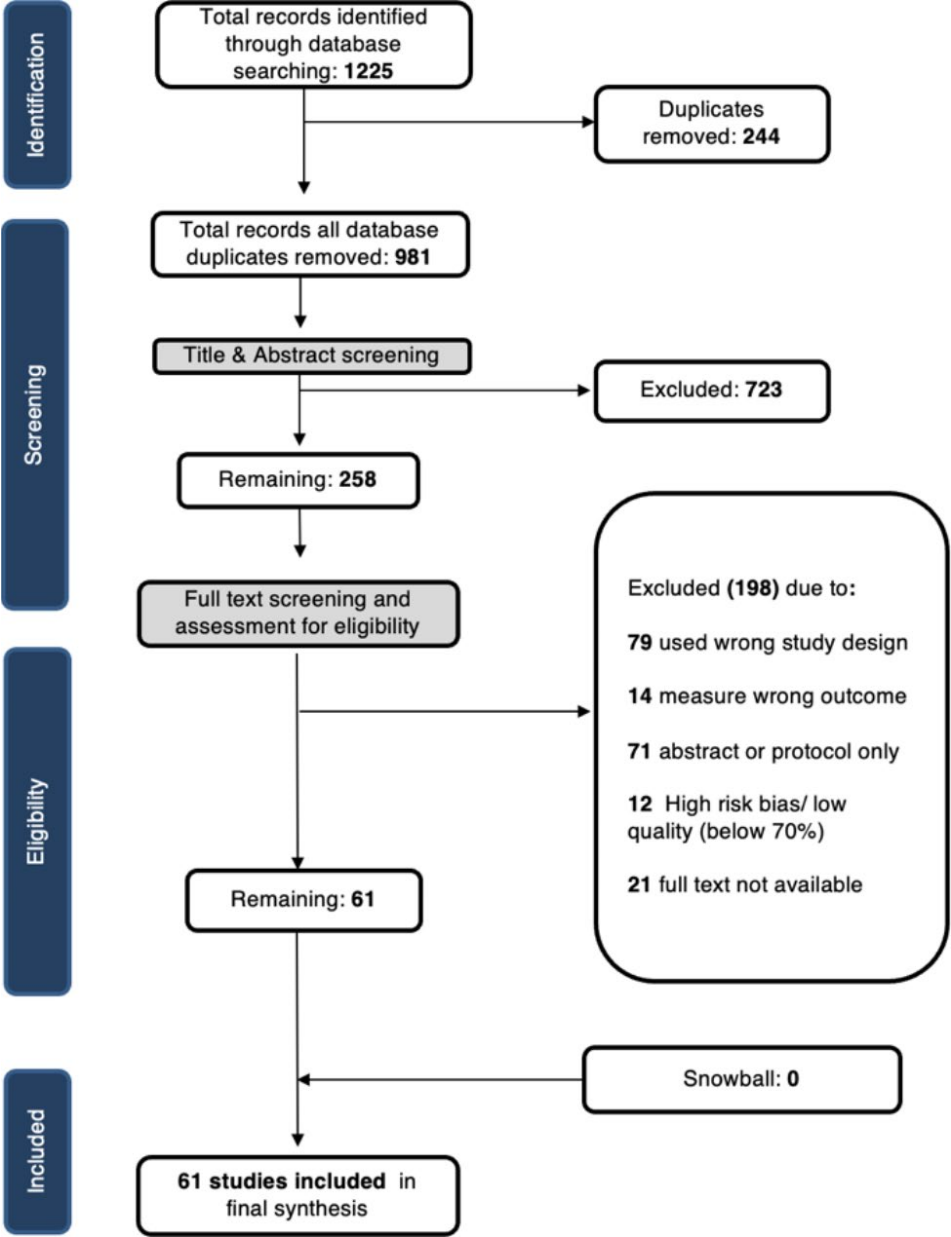
PRISMA Flow Diagram of Screening Process

Our initial search of the five databases identified 1,225 articles. Duplicates were removed, after which 981 titles and abstracts were screened by reviewers. We identified 723 studies for removal. The remaining 258 articles then underwent full-text review. Of these studies, 198 studies were excluded – 71 were published as abstracts only, 79 used a study design that did not meet our inclusion criterion (not randomized, controlled or quasi-experimental), 14 studies measured the irrelevant outcomes (not an objectively measured behavioural outcome). The full text was unavailable for 21 studies. Lastly, 12 studies were excluded due to poor quality (falling below the 70% threshold) as identified by the risk of bias assessment. The final analysis included 61 studies. Figure 1 presents a summary of our results.

Results are first reported by descriptive study characteristics. Using details and phrases in included papers, studies are then categorized by the mechanisms through which the interventions seek to modify patient behaviour; reminders, incentives/disincentives or other. This section then summarizes the comparative efficiency and characteristics of each delivery mode.

### Study characteristics

Full study characteristics of included articles are reported in Appendix 2. A majority of the studies were conducted in and used data from the United States (n = 28). Seven studies used data from the United Kingdom and five from Australia. Three studies were conducted in Switzerland, and another three were conducted in Malaysia. Two studies used data from Saudi Arabia. Two studies originated from Scotland specifically (not covering other regions in the United Kingdom). One study was conducted in and focused on Hong Kong specifically. Lastly, there was a single study from each of the following countries: Brazil, Cameroon, Canada, China, Denmark, Israel, South Korea, Nigeria, Pakistan, and the Netherlands.

The most commonly used study designs were randomised control study design (n = 41) and non-randomized experimental design (n = 15). The remaining studies used quasi-experimental approaches, controlling for variables in their analysis. Two studies utilised a cross-sectional study design, and one study used each of the following designs: cohort study, case-control study, and retrospective observational study.

Most studies included in their study populations anyone attending clinics (n = 43). However, ten studies defined their population as adult outpatient or primary care clinic patients, and seven studies focused on paediatric populations and their caretakers.

All studies assessed ambulatory or outpatient care services, although a variety of hospital departments or appointment types. Studies further focused on the following appointment types; primary care (n = 16), mental health (n = 8), gastrointestinal including patients scheduled for colonoscopies and endoscopies (n = 5), dental (n = 3), radiology/ diagnostic imaging (n = 2), addiction (n = 2) and one of each of the following, respiratory, physical therapy, radiation, emergency department, neurological exam, pain centre, eye clinic and paediatric HIV appointments.

Almost all studies in our review assessed non-financial interventions (n = 58). One study evaluated the use of financial penalties [23], and another used financial rewards to influence patient behaviour [24].

### Behaviour change interventions categories

We categorized the interventions by the mechanisms through which the interventions seek to modify patient behaviour – whether it is by addressing the heuristics or mental shortcuts that lead patients to make irrational or suboptimal choices. A summary of study intervention and effect size, organized by change intervention category is provided in Table 2. The most common behaviour change intervention assessed was the use of reminders (n = 56). Other categories included the use of incentives and disincentives and language and cultural congruency.

**Table 2:** Effect of interventions

Study	Country	Intervention	Findings (Odds Ratio or Co-efficient)
Reminders (n = 56)			
Arora 2015	US	SMS reminder	10.5% (CI 0.3 – 20.8%) *p* = 0.045
Arshad 2017	Pakistan	SMS reminder	OR 1.841 (CI 1.346–2.518)
Bigby 1983	US	Computer generated letter reminder and telephone reminder	24% vs. 14%
Bigna 2014	Cameroon	Text message and call reminder, text message reminder only or call reminder only	Text: OR 2.6 (CI 1.3–6.3) Call: OR 5.5 (CI 2.3–13.1) Text + call: OR 7.5 (CI 2.0–19.0)
Blaauw 2019	Netherlands	SMS reminder	-1.316 SD 0.015
Can 2003	UK	Reminder letter and confirmation slip	OR 0.43 (CI 0.19–0.96)
Chaiyachati 2018	US	Telephone reminder	36.5% vs. 36.7%
Chen 2018	China	Automated SMS reminders	28.8% (CI 17.9 – 39.8%) *p* < 0.001
Childers 2016	US	Telephone reminder	OR 0.67 (CI 0.50–0.91)
Chung 2020	US	Online portal for automated rescheduling of appointments and reminders	1.3% point (38%) reduction
Clough 2014	Australia	SMS reminders	No significant difference
Fairhurst 2008	UK	SMS reminders	OR 0.63 (CI 0.36–1.1)
Gerson 1986	US	Postcard or telephone reminders	
Griffin 2011	US	Interactive voice response (IVR) technology call reminder	No significant difference
Gullo 2018	Australia	SMS reminders	0.90 vs. 0.84 *p* = 0.02
Hallsworth 2015	UK	SMS reminders	OR 0.74 (CI 0.61–0.89)
Hashim 2001	US	Telephone reminder	0.19 *p* = 0.0065
JunodPerron 2013	Switzerland	Telephone and SMS reminders	OR 0.90 (CI 0.70–1.0)
Koren 1994	US	Postcard or telephone reminder	74.1% vs. 82.1% p < 0.001
Kourany 1990	US	Telephone reminder and letter describing first visit, telephone reminder phone call AND letter (4) no contact between initial call and scheduled appointment day	p < 0.03
Kravariti 2018	UK	SMS reminders	OR 2.95 (CI 1.05–8.85)
Krishna 2012	UK	Mailed reminders	OR 1.57 (CI 2.46–6.04)
Kwon 2012	South Korea	Telephone reminder	OR 0.07 (CI 0.01–0.61)
Lam 2021	China	SMS reminders	OR 0.72 (CI 0.54–0.95)
Lance 2021	Brazil	Telephone and SMS reminders	9.5% vs. 21% vs. 22.8% *p* = 0.025
Leong 2006	Malaysia	Telephone and SMS reminders	OR 1.59 (CI 1.17–2.17)
Liew 2009	Malaysia	Telephone or SMS reminders	SMS: OR 0.62 (CI 0.41–0.93) Telephone: OR 0.53 (CI 0.35–0.81)
Mahmud 2021	US	SMS reminders	53.1% vs. 54.4% *p* = 0.73
Mikhaeil 2019	Canada	Mailed reminder	7.1% vs. 6.3% *p* = 0.04
Milne 2010	UK		
Narring 2013	Switzerland	SMS reminders	20.0% vs. 16.4% *p* = 0.146
Nayor 2019	US	SMS and email reminders	OR 0.70 (CI 0.52–0.93)
Nelson 2011	US	SMS and email reminders	OR 2.12 (CI 1.03–4.38)
Parikh 2010	US	Telephone reminder	
Percac-Lima 2015	US	Telephone reminder	17.5% vs. 10.2% *p* < 0.001
Percac-Lima 2016	US	SMS reminders	19.8% vs. 18.0% *p* = 0.106
Perron 2010	Switzerland	Telephone and SMS reminders	11.4% vs. 7.8% *p* < 0.005
Quattlebaum 1991	US	Mailed computer generated reminders	19% vs. 10% *p* = 0.0002
Ritchie 2000	Australia	Telephone reminder	54.4% vs. 70.7% *p* = 0.002
Roberts 2007	UK	Telephone reminder	*p* = 0.036
Roseland 2022	US	Telephone and SMS reminders	CT *p* = 0.05 MRI *p* = 0.43
Ruggeri 2020	US	Telephone and SMS reminders	No significant effect
Rusius 1995	UK	Mailed reminder	28% vs. 13% *p* = 0.05
Senderey 2020	Israel	SMS reminders using various framings	Appointment cost: OR 0.72 (CI 0.68–0.77) Emotional relatives: OR 0.77 (CI 0.79–0.82) Emotional guilt OR 0.69 (CI 0.67–0.76) Social Norm: OR 0.73 (CI 0.61–0.79) Social Identify: OR 0.83 (CI 0.76–0.87)
Shah 2016	US	Telephone reminder	AR: -6.4% (CI -3.0% – -9.8%)
Sims 2012	UK	SMS reminders	7 and 5 days before appt: OR 1.75 (CI 1.37–2.23) 7 and 3 days before appt: OR 1.53 (CI 1.20–1.95)
Steiner 2016	US	Interactive voice response technology call reminder	OR 0.83 (CI 0.69–1.00)
Steiner 2018	US	Interactive voice response technology call reminder versus SMS reminders	1 day reminder: OR 0.93 (CI 0.84–1.04) 3- and 1-day reminders: OR 0. 75 (CI 0.67–0.84)
Stormon 2021	Australia	SMS reminders	Child FTA: IRR 1.04 (CI 0.99–1.09) Adult FTA: IRR 1.07 (CI 0.99–1.16)
Tan 2019	US	Automated SMS reminders	OR 6.77 (CI 5.45–8.41)
Taylor 2012	Australia	SMS reminders	OR 1.61 (CI 1.03–2.51)
Teeng 2021	Malaysia	SMS reminders	20% *p* = 0.002
Teo 2017	US	Live call reminder or voicemail message reminder	*p* = 0.35
Thomas 2017	Nigeria	SMS reminders	OR 1.8 (CI 1.02–3.19)
Youssef 2014	Saudi Arabia	SMS reminders	OR 0.56 (CI 0.28–0.82)
Youssef 2014	Saudi Arabia	SMS reminders	GM: OR 0.56 (CI 0.28–0.82) Neurology: OR 0.53 (CI 0.23–0.90) OBGYN: OR 1.00 (CI 0.71–1.42)
*Incentives and Disincentives (n = 2)*			
Blæhr 2018	Denmark	Fines for non-attendance	0.09% *p* = 0.0895
Lee 2020	US	$15 giftcard for attendance	OR 1.94 (CI 1.16–3.24)
*Other (n = 3)*			
Andreae 2017	US	Human reminder call in preferred language	RR 0.89 (CI 1.42–1.42)
Horvath 2011	US	Patient appointment portal, with email, telephone and SMS reminders	OR 1.39 (CI 1.22–1.57)
Groden 2021	US	Clinic signage and reminder appointment cards	59.5% vs. 74.3% *p* = 0.01

### Delivery mode of reminders

Reminders seek to steer patients’ attention towards particular decisions to create behaviour change. Heterogeneity in reminders was explored by categorising the variation between the mode of delivery and timing of reminders.

Studies assessed reminders delivered through three main channels short message system (SMS), text reminders or electronic reminders (n = 26), telephone (n = 13), or physical mail (n = 5). Thirteen studies assessed combinations of interventions, including phone and SMS (n = 8), telephone and mail (n = 4), or all three means of delivery mode (n = 1). One study delivered reminders via an online portal system [25] and another used clinic signage and appointment reminder cards [26].

The timing of reminders ranged from two hours to two weeks before a scheduled appointment. Most studies (n = 16) used reminders between one to three days before appointments; 15 studies focused employed short-term reminders (less than one week). Other common time ranges included one week (n = 6) and two weeks (n = 2). Nine studies varied the timing of reminders delivered in their interventions between one day to two weeks. There were eight studies where timing details were either not applicable to the intervention or not made available in the study.

**Fig. 2:**
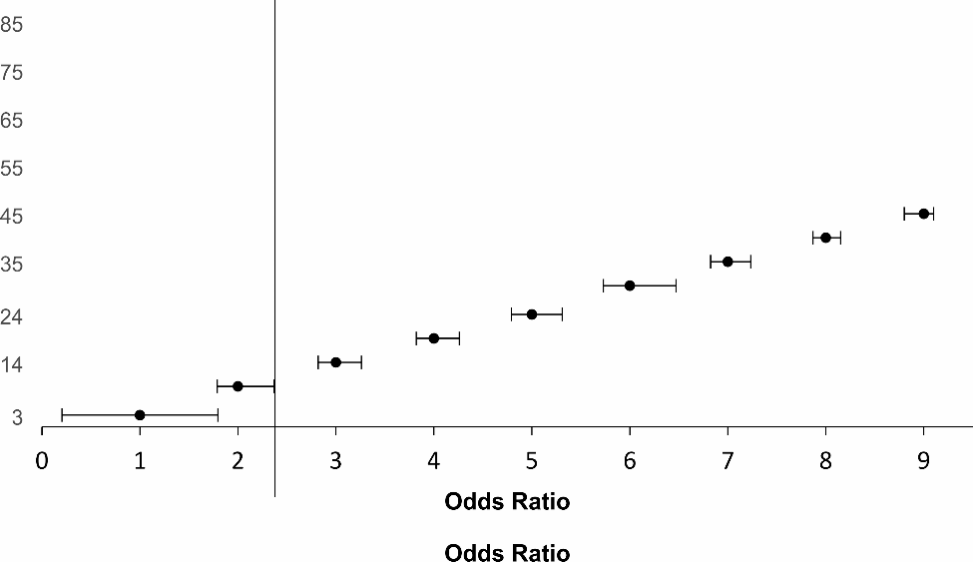
Effect of SMS/reminders on No-show rates

Overall, studies report mixed results on the effect of interventions involving reminders. Mailed reminders were found to increase kept appointments in all five studies [27–31]. A large number of studies (n = 15) found that using SMS reminders significantly reduced non-attendance rates. Simultaneously, four other studies using SMS reminders indicated that SMS reminders did not lead to reductions in non-attendance rates [32–35]. In another study, SMS reminders increased the number of unable to attend rates [36]. The effect size for studies that included odds ratios related to the effect of SMS reminders is reported in Fig. 2.

Interventions that relied on telephone calls to remind patients of their appointments yielded inconsistent results. One study found that telephone reminders may decrease no-show rates while simultaneously increasing patient cancellation rates [37]. Concomitantly, other studies found limited (null) effects and small coefficients and odds ratios of telephone reminder interventions on attendance [38, 39].

Figure 2 depicts the direction and magnitude of the overall effects on no-show rates across individual studies. An odds ratio above 1 shows that improvement in attendance for the SMS reminded group was greater than the non-reminded group. Using a random-effects meta-analysis model, our pooled summary effect of OR 1.21 (CI 0.41-2.00) indicates a positive effect of SMS reminders. Results of the Cochran’s Q test indicate no significant heterogeneity amongst the studies (Q = 7.5, p = 0.941) further confirmed by a the complementary Higgins I2 test (0%). The plot indicates that most of the reporting odds ratios pointed towards significant improvements in attendance for patients receiving SMS reminders over those who received none.

### Characteristics of reminder delivery mode interventions

A subgroup of these studies compared different ways to deliver reminders (e.g., SMS text compared to telephone, or automated telephone calls compared to calls provided by clinical staff) and reminder efficiency (n = 13).

Results were mixed regarding the most effective methods of delivering reminders. Although no-show rates did not vary significantly between specific reminders delivery methods (calls, letters, or receiving both), any type of contact was found to decrease no-show rates [40]. Patients receiving telephone calls were more likely to keep their appointments than those who received postcards [41]. Similar results were found when comparing phone calls to patients receiving SMS reminders [42–45]. Combining multiple methods of reminders, such as both text and calls, were found to be most effective under specific circumstances [46]. Hallsworth et al. found that reminder messages are more effective when the messages note the specific cost of a missed appointment to make the incurred costs of missed appointments more salient to the patients opportunity cost calculation [14].

Additionally, three studies compared the characteristics of telephone call reminders, testing the difference generated by the use of human-initiated calls versus automated calls. Results were mixed, where interactive voice response (IVR) system calls were as effective as real-life nurse phone calls [47] in some instances while clinic staff reminders were more effective in lowering no-show rates compared to automated reminder systems [48]. Additional studies found no meaningful change when switching to automated messaging from traditional human-initiated calls [49].

### Incentives / disincentives

Only two studies presented incentives or disincentives for patient behaviour [23, 24]. The change mechanism was most commonly associated with financial rewards or penalties for patients. Results of the studies indicate that incentives may be more successful that fines. One study found fining patients DKK250 (€34) for non-attendance did not appear to reduce non-attendance [23]. In another study, providing patients with small incentives to attend designated appointments, such as $15 gift cards to Target or CVS, was associated with improved appointment attendance [24]. The maximum pay out per patient was limited to $45 and patients in the study were 94% more likely to attend their appointments (OR 1.94 CI 1.16–3.24).

### Addressing opportunity costs

Opportunity costs, or the loss of potential benefits from other options when one option is chosen, also contribute to decisions patients make about attending appointments. Two studies assessed interventions, which sought to minimize opportunity costs for appointment attendance, including language and cultural congruency and transportation. Andreae 2017 et al. found that contacting patients using human reminder calls in patients’ preferred language before their appointment improved overall attendance rates [50]. Chaiyachati et al. combined patient reminders with an offer of free rideshare-based transportation services [51]. The authors found that offering transportation to patients may improve the convenience and reduce opportunity cost of attending appointments; however, ridesharing uptake was low and did not impact missed care appointments.

## Discussion

This systematic review described and summarized the published evidence on behavioural economic interventions to reduce patient non-attendance. We highlighted studies that point to the effectiveness of using behavioural economic interventions, such as reminders and financial incentives. In particular, our review identified a large body of literature related to the use of appointment reminders either via mail, telephone or SMS to improve patient attendance – all of which serves to nudge patients into attending their scheduled appointment. We found, mainly, that similar interventions and research have been repeated with minimal change over the past three decades. Namely, the studies focused on issuing reminders as interventions to reduce patient non-attendance. There has been minimal inclusion of additional mechanisms such as ways to remove barriers to care or rewards and/or penalties to incentivize attendance.

This section summarizes the strength of previous studies and underlines future research needs to add to our understanding of the behavioural economic mechanism and interventions that may reduce patient non-attendance in healthcare settings. Specifically, we address (1) the need to expand on the evaluation of the characteristics and mechanisms of the interventions implemented, (2) the sparse use of behavioural economic-based interventions and the necessity to understand behavioural issues related to non-attendance, and (3) the contemporaneous effect of interventions. We conclude our discussion with policy recommendations that can be derived from current findings.

***Detailed evaluation of characteristics and mechanisms of interventions.*** Reminders bring the appointment to the forefront of each patient’s thought process to circumvent attentional biases. This approach addresses the patients’ limitations in memory and attention, which may lead them to act against their self-interest of keeping appointment times. Of the studies which assessed the effectiveness of how reminders are delivered, ten reported mixed results or minimal changes. Subgroup analyses in one study found that changes in attendance rates varied between different consultation types and were significantly for general and smoking cessation consultations but insignificant in HIV clinics and dietician consultations [52]. Similarly, mixed results on outcomes were found in the case of reminders for electrodiagnostic examinations which lacked significance in attendance rate compared to the significant changes observed within only needle electromyography appointment attendance [39].

These findings underscore the criticality of analysing the characteristics as well as substantive content of reminder messages, preferably in conjuncture. We identified three studies that evaluated how the varying characteristics (e.g., automated calls or human-initiated calls) of reminders in the same mode (e.g., telephone calls) may impact the effectiveness of the intervention [47–49]. The results are mixed, with one study showing IVR calls were as effective as real-life nurse phone calls [47], one study indicating that reminder calls initiated by clinic staff reminders were more effective in lowering no-show rates [48], and the third finding substantively null results when switching to automated messaging from traditional human-initiated calls [49]. The mixed results may be due to an unobserved variable – the content of the messages. This finding suggests the content of the message may be the key to understanding the discrepancy in the above outlined effectiveness of reminders. This rationale is substantiated by one study findings that focused on how the framing of reminder messages impact the effectiveness of those messages [14, 53]. Senderey et al. tested eleven separate framings of SMS reminders, to drive different motivational narratives on appointment attendance. Five types of messages produced statistically significant effect of reducing no-show rates, with emotional guilt and specific cost message frames created the greatest difference in no-show rates [53]. Furthermore, reminders in patients preferred language were particularly effective amongst Hispanic patients, pointing to the success of the cultural congruence [50].

***Behavioural economic-based interventions.*** Our review identified significant evidence supporting the effectiveness of reminders (either by SMS, telephone, or mail) across various settings. However, there is a lack of evidence regarding alternative interventions and efforts to address other heuristics, leaving a majority of behavioural economic approaches unused and unassessed. For example, in one study – excluded in our review due to the high risk of bias – patient choice and agency were employed in interventions that allowed patients to select their initial appointment dates and times [54]. To determine how to leverage behavioural economics in ameliorating patient non-attendance, it is crucial to first discuss behavioural economic issues in appointment attendance.

High opportunity costs, such as costly or inconvenient transportation, or the need for a translator, can contribute to increased DNA rates. Removing or mitigating opportunity costs can be seen as a way to reduce barriers to care, increasing the likelihood that patients honour their scheduled appointment. Patients may be more likely to miss appointments if consequences for non-attendance are low, or obligation to attend is not in the forefront. The broad body of evidence points to the value of financial penalties, if they are executed correctly [18, 55, 56]. Other approaches such as loss incentives (penalties), or gain incentives (rewards) are theoretically promising and should be explored in future research. The lack of richness to the variety of available behavioural economic approaches found in our review could be due to the relative novelty of behavioural economics in public policymaking. Popularized for use in public policy during the early 2000’s, and furthered by the widespread recognition of the importance of the “nudge theory” introduced by Sunstein and Reich in 2017 [57], enough time may not have passed to allow for a significant body of robust randomized controlled trials to test these theories.

***Contemporaneous effects of interventions.*** Our review identified six studies which assessed mail-based reminders; all but one was published prior to 2012. We reason that the ubiquitous nature of smartphone use and the estimated 67.1% penetration rate of the present day [58] may make physically mailed reminders a less cost-effective strategy when compared to SMS or phone calls. While we did not set a time frame for study inclusion, as we reason that the issue of non-attendance and the intervention to reduce non-attendance is a timeless one, we want to highlight that time-period and context may make one effective strategy more or less cost-effective when compared to other strategies. However, despite the demonstrated effectiveness of physically mailed reminders, in the current global context, we would recommend a careful evaluation of the strategy and the healthcare context prior to adopting sending reminders through the channel of physical mails.

***Future Studies and Policy Recommendations.*** Many studies in our review were conducted in public settings where payments are not required for patient’s visits. Evidence points to individuals being motivated by losses more than gains [59]. Using this knowledge to craft loss incentives, such as has been proven successful in other areas of public health concern, including increasing physical activity amongst obese adults [60]. Another approach is no-show fees to incentivize patient attendance. We recognize that the broader evidence indicates that penalties may be problematic and exacerbate disparities in healthcare [61–63]; however, we reason that this approach may be beneficial for patients from all background as well as cost-effective at a societal level when combined with other interventions such as a reminder system. Although the body of evidence is limited in our review, there are some indications that positive financial incentives could have a stronger impact that negative ones [23, 24].

Patients are prone to present bias in which the benefits of the care received from attending an appointment, particularly for chronic or primary care visits, occurs in the distant future. The benefits of the visit may offer long-term benefits, however this might come into direct conflict with the immediate costs of attending the appointment, such as missed working hours or transportation costs. Lowering opportunity costs for patients may be another effective approach that warrants further evaluation. The opportunity costs of adults seeking medical care through ambulatory services have been estimated at $43, which was substantial and exceeds the average patient’s out of pocket payment [64]. Although there was minimal evidence available in our review in regards to interventions addressing opportunity costs, broader literature points to the value of minimizing opportunity costs to improve health care delivery. For example, Lee et al. 2020 found that it is possible that the study setting of a safety-net based primary care hospital contributed to the strength of effectiveness of the incentive based intervention [24]. The small financial benefit may have a particularly strong effect on lowering opportunity costs for patients most at risk. Furthermore, these interventions could not only increase health system efficiency but also result in high patient satisfaction.

The bulk of our evidence assessed the use of reminders to address barriers to access. Nevertheless, existing public health literature has robust evidence supporting the pattern that mechanisms that can reduce the barriers to care results in increased healthcare utilization [65–67]. One potential intervention is leveraging heuristics to incentivize patients to circumvent these barriers and access care. For example, offering a one-time fee waiver if patients were to sign up for an online portal that can help patients schedule telehealth care when patients cannot make it to in-person care. Another intervention may be offering incentives for patients to sign up for a ride-share service, making it easier for patients to attend appointments while incurring a lower transportation cost. Future studies must endeavour to better understand the heuristics that lead patients to engage in behaviours that minimize the difficulties of attending an appointment.

Lastly, four studies included in our review discussed the cost or cost-effectiveness of these interventions [27, 68–70]. This is a good start; however, cost and budget impact assessments will be a critical part of any decision-maker’s choice to implement the interventions assessed in this review. Therefore, further research into the cost implications of any behavioural economic interventions, especially in comparison to the potential losses faced by high no-show rates, should be prioritized in any future research.

### Limitations

Our review has several important limitations. Firstly, some studies utilized interventions and quality study design that met the inclusion criteria, but did not report outcomes in a manner that allowed the pooling of results in a meta-analysis, were consequently excluded from this review. As we aim to include comparable results, many studies were excluded in the risk of bias assessment step due to a lack of study design that enables causal inference; however, many of these studies included unique interventions that warrant future evaluation. Because our search terms included “behavioural economics”, some studies that included intervention(s) that are behavioural economics-oriented and incentivize desired behaviour may have been excluded if the authors did not categorize their intervention as one that derives from behavioural economics. Lastly, we are cognizant that publication bias may favour studies with positive results, leaving out many interventions to reduce patient non-attendance; nevertheless, our review captures ten studies with null results [23, 32–36, 39, 49, 51, 68].

## Conclusion

Our review identified 61 studies on the use of behavioural economic interventions to reduce no-show rates. The included studies reflect a lack of diversity in intervention approaches but point to the effectiveness of reminder systems in reducing no-show rates across a variety of medical departments. We recommend future studies to test additional behavioural economic interventions that have not been used, tested, and/or published before. And, when examining frequently tested interventions, such as reminders, one should focus on the substantive aspect of the reminder message (e.g., framing of the message) and the characteristics of these messages (e.g., automated or human-initiated). Decision-makers will want to consider current findings with caution and ensure to evaluate the healthcare context before implementing effective interventions outlined by this systematic review.

### Electronic supplementary material

Below is the link to the electronic supplementary material.

Supplementary Material 1

Supplementary Material 2

Supplementary Material 3

## Data Availability

All data generated or analyzed during this study are included in this published article and its supplementary information file.
