# Supplementary material for: Behavioural economic interventions to reduce health care appointment non-attendance: a systematic review and meta-analysis
Source: BMC Health Serv Res. 2023 Oct 23;23:1136. doi: 10.1186/s12913-023-10059-9 (PMC10594857; doi:10.1186/s12913-023-10059-9)
Supplement: Supplementary file 1 — Supplementary Material 1 [file 12913_2023_10059_MOESM1_ESM.docx]

Supplementary 1. Database Search Strategy

| **Database** | **Search strings** |
| --- | --- |
| PubMed | (Appointments and Schedules OR Appointments, Patient OR schedules, patient [MeSH] OR “schedules and appointments” OR “schedules” OR “schedule” OR “patient schedules” OR “patient schedule” OR “schedule, patient” OR “appointments” OR “appointment” OR “patient appointments” OR “patient appointment” OR “medical appointment”) AND (No-show patients [MeSH] OR “no show” OR “no-show” OR “non-attendance” OR “missed appointment*” OR “fail to attend” OR “failed to attend” OR “cancell*” ) AND (Economics, Behavioral [MeSH] OR “financial incentive” OR “financial penalt*” OR “fine” OR “penalt*” OR “monetary sanction” OR “behavioural economics” OR “behavioral economics” OR “asymmetric paternalism” OR “nudg*” OR “choice architect*” OR “reframe” OR “loss aversion” OR “endowment” OR “prospect theory” OR “feedback” OR “social comparison” OR “social norm” OR “active choice” OR “prompted choice” OR “accountable justification” OR “suggested alternative” OR “mental accounting” OR “allocation bias” OR “reminders” OR “salience” OR “commit*” OR “precommitment”) |
| Embase | ‘appointments and schedules’/exp OR ‘appointments, patient’/exp OR ‘schedules, patient’/exp OR (‘schedules and appointments’ OR ‘schedule*’ OR ‘patient schedule*’ OR ‘appointment*’ OR ‘patient appointment*’ OR ‘medical appointment*’):ti,ab,kw  AND  ‘No-show patients’/exp OR (‘no show’ OR ‘no-show’ OR ‘non-attendance” OR ‘missed appointment*’ OR ‘fail to attend’ OR ‘failed to attend’ OR ‘cancell*’ OR ‘nonattend*’ OR ((missed OR misses OR missing) NEAR/3 (visit OR visits OR appointment*))):ti,ab,kw  AND  ‘Economics, Behavioral’/exp OR (((‘financial’)NEAR/2 (incentive OR penalt*)) OR ‘fine’ OR ‘penalt*’ OR ‘monetary sanction’ OR ‘behavioural economic*’ OR ‘behavioral economic*’ OR ‘asymmetric paternalism’ OR ‘nudg*’ OR ‘choice architect*’ OR ‘reframe’ OR ‘loss aversion’ OR ‘endowment’ OR ‘prospect theory’ OR ‘feedback’ OR ‘social comparison’ OR ‘social norm’ OR ‘active choice’ OR ‘prompted choice’ OR ‘accountable justification’ OR ‘suggested alternative’ OR ‘mental accounting’ OR ‘allocation bias’ OR ‘reminder*’ OR ‘salience’ OR ‘commit*’ OR ‘precommitment’):ti,ab,kw |
| Web of Science | 5 #4 NOT DT=(Note OR Letter OR Meeting Abstract OR News Item OR Editorial Material)  4 #1 AND #2 AND #3  3 ALL = (“financial incentive” OR “financial penalty” OR “financial penalties” OR “fine” OR “penalty” OR “penalties” OR “monetary sanction” OR “behavioural economics” OR “behavioral economics” OR “asymmetric paternalism” OR “nudge” OR “choice architect” OR “choice architecture” OR “reframe” OR “loss aversion” OR “endowment” OR “prospect theory” OR “feedback” OR “social comparison” OR “social norm” OR “active choice” OR “prompted choice” OR “accountable justification” OR “suggested alternative” OR “mental accounting” OR “allocation bias” OR “reminders” OR “salience” OR “commitment” OR “commit” OR “precommitment”)  2 ALL = (“no-show patients” OR “no show” OR “no-show” OR “non-attendance” OR “missed appointment*” OR “fail to attend” OR “missed appointment” OR “missed visit” OR “missing appointment” OR “missing visit” OR “failed to attend” OR “canceled” OR “cancel”)  1 ALL = (“schedules” OR “schedule” OR “patient schedules” OR “patient schedule” OR “appointments” OR “appointment” OR “patient appointments” OR “patient appointment” OR “medical appointment”) |
| Cochrane | #1 MeSH descriptor: [Appointments and Schedules] explode all trees  #2 (“schedules and appointments” OR “schedules” OR “schedule” OR “patient schedules” OR “patient schedule” OR “schedule, patient” OR “appointments” OR “appointment” OR “patient appointments” OR “patient appointment” OR “medical appointment”):ti,ab,kw  #3 #1 OR #2  #4 MeSH descriptor: [No-Show Patients] explode all trees  #5 (“no show” OR “no-show” OR “non-attendance” OR “missed appointment*” OR “fail to attend” OR “failed to attend” OR “cancell*”):ti,ab,kw  #6 #4 OR #5  #7 MeSH descriptor: [Economics, Behavioral] explode all trees  #8 (“financial incentive” OR “financial penalt*” OR  “fine” OR “penalt*” OR “monetary sanction” OR “behavioural economics” OR “behavioral economics” OR “asymmetric paternalism” OR “nudg*” OR “choice architect*” OR “reframe” OR “loss aversion” OR “endowment” OR “prospect theory” OR “feedback” OR “social comparison” OR “social norm” OR “active choice” OR “prompted choice” OR “accountable justification” OR “suggested alternative” OR “mental accounting” OR “allocation bias” OR “reminders” OR “salience” OR “commit*” OR “precommitment”):ti,ab,kw  #9 #7 OR #8  #10 #3 AND #6 AND #9 |
