# Supplementary material for: Behavioural economic interventions to reduce health care appointment non-attendance: a systematic review and meta-analysis
Source: BMC Health Serv Res. 2023 Oct 23;23:1136. doi: 10.1186/s12913-023-10059-9 (PMC10594857; doi:10.1186/s12913-023-10059-9)
Supplement: Supplementary file 2 — Supplementary Material 2 [file 12913_2023_10059_MOESM2_ESM.docx]

Appendix 1. Risk Bias Assessment

|  | **Screening** | | **Quantitative randomized controlled trials** | | | | | **Quantitative non-randomized** | | | | |  |
| --- | --- | --- | --- | --- | --- | --- | --- | --- | --- | --- | --- | --- | --- |
| **Author (Year) [Reference]** | *Are there clear research questions?* | *Do the collected data allow to address the research questions?* | *Is randomization appropriately performed?* | *Are the groups comparable at baseline?* | *Are there complete outcome data?* | *Are outcome assessors blinded to the intervention provided?* | *Did the participants adhere to the assigned intervention?* | *Are the participants representative of the target population?* | *Are measurements appropriate regarding both the outcome and intervention (or exposure)?* | *Are there complete outcome data?* | *Are the confounders accounted for in the design and analysis?* | *During the study period, is the intervention administered (or exposure occurred) as intended?* | **TOTAL SCORE (OUT OF 7)** |
| Andreae et al. (2017)[44] | Y | Y | Y | Y | Unclear | Y | Y |  |  |  |  |  | 6 |
| Arora et al. (2015)[63] | Y | Y | Y | Y | Unclear | Y | Y |  |  |  |  |  | 6 |
| Arshad (2017)[64] | Y | Y | Y | Y | Unclear | Unclear | Y |  |  |  |  |  | 5 |
| Bigby et al. (1983)[65] | Y | Y | Y | Y | Unclear | Unclear | Y |  |  |  |  |  | 5 |
| Bigna et al. (2014)[41] | Y | Y | Y | Y | Y | Y | Y |  |  |  |  |  | 7 |
| Blaauw et al.(2019)[66] | Y | Y | . | . | . | . |  | Y | Y | Unclear | Y | Y | 6 |
| Blæhr et al. (2018)[15] | Y | Y | Y | Y | Y | Y | Y |  |  |  |  |  | 7 |
| Can et al. (2003)[30] | Y | Y | Y | Y | Unclear | Unclear | Y |  |  |  |  |  | 5 |
| Chaiyachati et al. (2018)[21] | Y | Y |  |  |  |  |  | Y | Y | Unclear | Y | Y | 6 |
| Chen et al. (2018)[67] | Y | Y | Y | Y | Unclear | Unclear | Y |  |  |  |  |  | 5 |
| Childers et al. (2016)[68] | Y | Y |  |  |  |  |  | Y | Y | Unclear | Y | Y | 6 |
| Chung et al. (2020)[26] | Y | Y |  |  |  |  |  | Y | Y | N | Y | Y | 6 |
| Clough & Casey (2014)[20] | Y | Y | Y | Y | Unclear | Unclear | Y |  |  |  |  |  | 5 |
| Fairhurst & Sheikh (2008)[16] | Y | Y | Y | Y | Unclear | Unclear | Y |  |  |  |  |  | 5 |
| Flower et al. (2020)[69] | Y | N |  |  |  |  |  | Y | Y | Unclear | N | Y | 4 |
| Foley & O'Neill (2009)[70] | Y | N |  |  |  |  |  | Y | Y | Unclear | N | Y | 4 |
| Gerson et al. (1986)[71] | Y | Y | Y | Y | Y | Unclear | Y |  |  |  |  |  | 6 |
| Griffin et al. (2011)[42] | Y | Y | Y | Y | Y | Y | Y |  |  |  |  |  | 7 |
| Groden et al. (2021)[27] | Y | Y |  |  |  |  |  | Y | Y | Unclear | N | Y | 5 |
| Gullo et al. (2018)[72] | Y | Y |  |  |  |  |  | Y | Y | Y | Y | Y | 7 |
| Hallsworth et al. (2015)[6] | Y | Y | Y | Y | Unclear | Unclear | Y |  |  |  |  |  | 5 |
| Hashim et al. (2001)[33] | Y | Y | Y | Y | Unclear | Unclear | Y |  |  |  |  |  | 5 |
| Horvath et al. (2011)[73] | Y | Y |  |  |  |  |  | Y | Y | Unclear | N | Y | 5 |
| Jenkins (2017)[47] | Y | N |  |  |  |  |  | Y | Y | Unclear | N | Y | 4 |
| Jeppesen & Ainsworth (2015)[74] | Y | N |  |  |  |  |  | Y | Y | Unclear | N | Y | 4 |
| Junod Perron et al. (2013) [17] | Y | Y | Y | Y | Y | Y | Y |  |  |  |  |  | 7 |
| Koren et al. (1994)[37] | Y | Y | Y | Y | Unclear | N | Y |  |  |  |  |  | 5 |
| Kourany et al. (1990)[40] | Y | Y | Y | Y | Y | Unclear | Y |  |  |  |  |  | 6 |
| Kovach & Flores(2021)[75] | Y | N |  |  |  |  |  | Y | N | Unclear | N | Y | 3 |
| Kravariti et al. (2018)[76] | Y | Y | Y | Y | Unclear | Unclear | Y |  |  |  |  |  | 5 |
| Krishna & Amarjothi (2012)[31] | Y | Y | Y | Y | Y | Unclear | Y |  |  |  |  |  | 6 |
| Kunigiri et al.(2014)[77] | Y | N | Unclear | Y | Unclear | N | Y |  |  |  |  |  | 3 |
| Kwon et al. (2012)[18] | Y | Y |  |  |  |  |  | Y | Y | Y | N | Y | 6 |
| Lam et al (2021)[78] | Y | Y | Y | Y | Y | Y | Y |  |  |  |  |  | 7 |
| Lance et al. (2021)[38] | N | Y |  |  |  |  |  | Y | Y | Y | Y | Y | 6 |
| Lee et al. (2020)[25] | Y | Y |  |  |  |  |  | Y | Y | Y | Y | Y | 7 |
| Leong et al (2006)[35] | Y | Y | Y | Y | Y | Unclear | Y |  |  |  |  |  | 6 |
| Liew et al. (2009)[39] | Y | Y | Y | Y | Y | N | Y |  |  |  |  |  | 6 |
| MacLean et al. (1989)[79] | Y | Y | Unclear | Unclear | Y | N | Y |  |  |  |  |  | 4 |
| Mahmud et al. (2021)[22] | Y | Y | Y | Y | Y | Y | Y |  |  |  |  |  | 7 |
| Mikhaeil et al. (2019)[32] | Y | Y |  |  |  |  |  | Y | Y | Y | N | N | 5 |
| Milne (2010)[80] | Y | Y |  |  |  |  |  | Y | Y | Y | Unclear | Y | 6 |
| Narring et al. (2013)[19] | Y | Y | Y | Y | Y | Y | Y |  |  |  |  |  | 7 |
| Nayor et al. (2019)[81] | Y | Y |  |  |  |  |  | Y | Y | Y | Y | Y | 7 |
| Nelson et al. (2011)[36] | Y | Y | Y | Y | Y | N | Y |  |  |  |  |  | 6 |
| Parikh et al. (2010)[43] | Y | Y | Y | Y | Y | N | Y |  |  |  |  |  | 6 |
| Percac-Lima et al. (2015)[82] | Y | Y | Unsure | Y | Y | N | Y |  |  |  |  |  | 5 |
| Percac-Lima et al. (2016)[83] | Y | Y | Unsure | Y | Y | N | Y |  |  |  |  |  | 5 |
| Perron et al. (2010)[45] | Y | Y | Y | Y | Y | N | Y |  |  |  |  |  | 6 |
| Prasad & Anand (2012)[84] | Y | Y | N | Unclear | Y | N | Y |  |  |  |  |  | 4 |
| Quattlebaum et al. (1991)[28] | Y | Y |  |  |  |  |  | Y | Y | Y | N | Y | 6 |
| Reti (2003)[85] | Y | Y | N | Unclear | Y | N | Y |  |  |  |  |  | 4 |
| Ritchie et al. (2000)[86] | Y | Y | Y | Y | Y | N | Y |  |  |  |  |  | 6 |
| Roberts et al. (2007)[62] | Y | Y | Y | Y | Y | N | Y |  |  |  |  |  | 6 |
| Roseland et al. (2022) [24] | Y | Unsure |  |  |  |  |  | Y | Y | Y | Unclear | Y | 5 |
| Ruggeri et al. (2020)[34] | Y | Y |  |  |  |  |  | Y | Y | Y | Y | Y | 7 |
| Rusius (1995)[29] | Y | Y | Unclear | Unclear | Y | Y | Y |  |  |  |  |  | 5 |
| Sawyer et al. (2002)[87] | Y | Y | Unclear | Unclear | Y | N | Y |  |  |  |  |  | 4 |
| Senderey et al. (2020)[46] | Y | Y | Y | Unclear | Y | N | Y |  |  |  |  |  | 5 |
| Shah et al. (2016)[88] | Y | Y | Y | Unclear | Y | N | Y |  |  |  |  |  | 5 |
| Sims et al. (2012)[61] | Y | Y |  |  |  |  |  | Y | Y | Y | Y | Y | 7 |
| Steiner et al. (2016)[89] | Y | Y | Y | Y | Y | N | Y |  |  |  |  |  | 6 |
| Steiner et al. (2018)[90] | Y | Y | Y | Y | Y | N | Y |  |  |  |  |  | 6 |
| Stormon et al.(2021)[23] | Y | Y |  |  |  |  |  | Y | Y | Y | Unclear | Y | 6 |
| Tan et al.(2019)[91] | Y | Y |  |  |  |  |  | Y | Y | Y | Y | Y | 7 |
| Taylor et al. (2012)[92] | Y | Y | Y | Y | Y | Y | Y |  |  |  |  |  | 7 |
| Teeng et al. (2021)[93] | Y | Y | Y | Y | Y | N | Y |  |  |  |  |  | 6 |
| Teo et al. (2017)[94] | Y | Y |  |  |  |  |  | Unclear | Y | Y | Y | Y | 6 |
| Thomas et al. (2017)[95] | Y | Y | Y | Unclear | Y | Y | Y |  |  |  |  |  | 6 |
| Turner & Vernon (1976)[96] | Y | Unclear |  |  |  |  |  | Y | Y | Y | Unclear | Unclear | 4 |
| Youssef (2014)[97] | Y | Y | Y | Y | Y | Unclear | Y |  |  |  |  |  | 6 |
| Youssef et al. (2014)[98] | Y | Y | Y | Y | Y | Y | Y |  |  |  |  |  | 7 |

Appendix 2. Included Studies Attributes

| **Author (Year)** | **Title** | **Descriptive characteristics** | **Intervention details** | **Results** |
| --- | --- | --- | --- | --- |
| Andreae et al. (2017)[44] | A pragmatic trial to improve adherence with scheduled appointments in an inner-city pain clinic by human phone calls in the patient's preferred language | **Intervention/ comparator:** human reminder call in preferred language / no phone contact  **Hospital dept:** Pain center  **Population (n):** Adults with scheduled appointments (963)  **Country**: USA  **Study design:** Randomised controlled trial | **Mechanism:** Language and Cultural congruency, non-financial  **Time/freq:** 1 day prior | **Results:** Human phone reminders in the preferred language increased adherence (RR 1.89, CI95% [1.42, 1.42], (pb0.01).  **Author's conclusion:** The intervention seemed particularly effective in Hispanic patients, supporting our hypothesis of cultural congruence as possible underlying mechanism. |
| Arora et al. (2015)[63] | Improving attendance at post-emergency department follow-up via automated text message appointment reminders: A randomized controlled trial | **Intervention/ comparator**: Automated text message reminder / no SMS  **Hospital dept:** Emergency dept  **Population (n):** Patients with text-capable mobile phones (374)  **Country**: USA  **Study design:** Randomised controlled trial | **Mechanism**: Reminder, non-financial  **Time/freq**: 7, 3 and 1 days prior | **Results:** The overall appointment adherence rate was 72.6% in the intervention group compared with 62.1% in the control group (difference between groups=10.5%, 95% confidence interval [CI]=0.3% to 20.8%; p=0.045; number needed to treat=9.5)  **Author's conclusion:** Automated text message appointment reminders resulted in improvement in attendance at scheduled post-ED discharge outpatient follow-up visits and represent a low-cost and highly scalable solution to increase attendance at post-ED follow-up appointments |
| Arshad (2017)[64] | Use of text reminders to reduce no-shows in an internal medicine outdoor clinic | **Intervention/ comparator:** SMS reminder /no SMS  **Hospital dept:** General medicine outdoor clinic  **Population (n):** General clinic patients (641)  **Country**: Pakistan  **Study design:** Randomised controlled trial | **Mechanism**: Reminder, non-financial  **Time/freq**: 1 day prior | **Results:** Patients send SMS were 1.8 times more likely to keep their appointments  **Author's conclusion:** Text messages improve no-show rates in general medicine clinics. |
| Bigby et al. (1983)[65] | Appointment reminders to reduce no-show rates. A stratified analysis of their cost-effectiveness | **Intervention/ comparator:**  Computer generated letter reminder and telephone reminder / no contact  **Hospital dept:** Adult primary care internal medicine  **Population (n):** All patients cared for by physicians and scheduled for follow-up (590)  **Country**: USA  **Study design:** Randomised controlled trial | **Mechanism**: Reminder, non-financial  **Time/freq**: 2 weeks prior | **Results:** The no-show rate was reduced from 24% in the control group to 14% in the reminder group; letter and telephoned reminders were equally effective  **Author's conclusion:** Telephoned or manual letter reminders should be cost-effective in many other ambulatory settings. |
| Bigna et al. (2014)[41] | Effect of mobile phone reminders on follow-up medical care of children exposed to or infected with HIV in Cameroon (MORE CARE): A multicentre, single-blind, factorial, randomised controlled trial | **Intervention/ comparator**: SMS and call reminders/ no reminder  **Hospital dept:** HIV appointments  **Population (n):** Children infected with or exposed to HIV (242)  **Country**: Cameroon  **Study design:** Randomised controlled trial | **Mechanism**: Reminder, non-financial  **Time/freq**: 2-3 days prior | **Results:**  **Author's conclusion:** **T**hee most effective mobile-phone-based method to improve the proportion of paediatric patients attending HIV appointments is the combined use of text messages and phone calls (with no synergistic effect between the two). |
| Blaauw et al.(2019)[66] | The Influence of a Short Message Service Reminder on Non-Attendance in Addiction Care | **Intervention/ comparator:** SMS reminder/ no reminder  **Hospital dept:** Addiction  **Population (n):** Outpatient patients (12797)  **Country**: The Netherlands  **Study design:** Cohort study | **Mechanism**: Reminder, non-financial  **Time/freq**: n/a | **Results:** Non-attendance was statistically significantly lower for appointments of patients who had received an SMS reminder (20.5%) than for appointments of patients who had not received a reminder (21.9%)  **Author's conclusion:** Sending an SMS reminder is associated with a statistically significant lower non-attendance at appointments by patients with a substance use disorder, but the differences have hardly any clinical significance |
| Blæhr et al. (2018)[15] | Effectiveness and cost-effectiveness of fining non-attendance at public hospitals: A randomised controlled trial from Danish outpatient clinics | **Intervention/ comparator:** Fine of €34 for non attendance/ no fine  **Hospital dept:** Orthopaedics  **Population (n):** First appointment attendees to clinic **(**6746)  **Country**: Denmark  **Study design:** Randomised controlled trial | **Mechanism**: Fines, financial (penalty)  **Time/freq**: n/a | **Results:** Of the 3333 appointments randomised to the fine policy, 130 (5%) of non-cancelled appointments were unattended, and of the 3413 appointments randomised to no-fine policy, 131 (5%) were unattended.  **Author's conclusion:** At a baseline level of around 5%, fining non-attendance does not seem to further reduce non-attendance. |
| Can et al. (2003)[30] | The use of postal reminders to reduce non-attendance at an orthodontic clinic: a randomised controlled trial. | **Intervention/ comparator:** Reminder letter and return confirmation slip/ no reminder  **Hospital dept:** Dentistry  **Population (n):** Patients attending clinic (232)  **Country**: UK  **Study design:** Randomised controlled trial | **Mechanism**: Reminder, non-financial  **Time/freq**: 2 weeks prior | **Results:**  **Author's conclusion:** The use of postal reminders for orthodontic consultation appointments appears to result in a useful increase of appointments that are kept or cancelled in advance. |
| Chaiyachati et al. (2018)[21] | Association of rideshare-based transportation services and missed primary care appointments: A clinical trial | **Intervention/ comparator:**  additional rideshare offer/ three appointment reminder phonecalls  **Hospital dept:** Primary care  **Population (n):** Medicaid beneficiaries (786)  **Country**: USA  **Study design:** Non-randomised experimental study | **Mechanism**: Reminder, non-financial  **Time/freq**: 2 days prior | **Results:**  **Author's conclusion:** Uptake of ridesharing was low and did not decrease missed primary care appointments. |
| Chen et al. (2018)[67] | A Mobile Phone Informational Reminder to Improve Eye Care Adherence Among Diabetic Patients in Rural China: A Randomized Controlled Trial | **Intervention/ comparator:** SMS reminder/ no reminder  **Hospital dept:** Eye clinic  **Population (n):** Diabetic patients attending eye clinic (230)  **Country**: China  **Study design:** Randomised controlled trial | **Mechanism**: Reminder, non-financial  **Time/freq**: 10 days prior | **Results:** Attendance for the Intervention group (51/119, [42.9%]) was significantly higher than for Controls (16/114, [14.0%],between-group difference 28.8% [95% (CI) 17.9%, 39.8%],P<.001).  **Author's conclusion:** Low-cost SMS informational reminders significantly improved adherence to, knowledge about, and satisfaction with care. |
| Childers et al. (2016)[68] | The role of a nurse telephone call to prevent no-shows in endoscopy | **Intervention/ comparator:** Telephone call/ no call  **Hospital dept:** Endoscopy/ Colonoscopy  **Population (n):** Patients scheduled for upper endoscopy or colonoscopy (2447)  **Country**: USA  **Study design:** Non-randomised experimental study | **Mechanism**: Reminder, non-financial  **Time/freq**: 1 week prior | **Results:** nurse phone call was associated with a 33% reduction in the odds of a no-show visit (odds ratio, 0.67; 95% confidence interval, 0.50-0.91)  **Author's conclusion:** Endoscopy practices may increase revenue, improve scheduling efficiency, and maximize resource utilization by hiring a nurse to reduce no-shows. |
| Chung et al. (2020)[26] | Patient-centric scheduling with the implementation of health information technology to improve the patient experience and access to care: Retrospective case-control analysis | **Intervention/ comparator:** Fast pass user/ non user  **Hospital dept:** General outpatient  **Population (n):** Patients who opt into online patient portal (14717)  **Country**: USA  **Study design:** Cross sectional study | **Mechanism**: Reminder, non-financial  **Time/freq**: n/a | **Results:** There was a 1.3 percentage point (38%) reduction in no-show rates among Fast Pass accepted appointments compared to other appointments with matching characteristics (P<.001).  **Author's conclusion:** An automated rescheduling system can improve patients’ access by reducing wait times for an appointment, with an added benefit of reducing no-shows by serving as a reminder of an upcoming appointment. |
| Clough & Casey (2014)[20] | Using SMS reminders in psychology clinics: a cautionary tale | **Intervention/ comparator:** SMS reminder/ no reminder  **Hospital dept:** Mental health  **Population (n):** Adult clients seeking psychotherapeutic treatment (140)  **Country**: Australia  **Study design:** Randomised controlled trial | **Mechanism**: Reminder, non-financial  **Time/freq**: 1 day prior | **Results:** No significant differences were found between the SMS and no SMS conditions **in relation to appointment attendance.**  **Author's conclusion:** SMS appointment reminders were not effective at increasing appointment attendance. |
| Fairhurst & Sheikh (2008)[16] | Texting appointment reminders to repeated non-attenders in primary care: Randomised controlled study | **Intervention/ comparator:** SMS reminder/ no reminder  **Hospital dept:** Primary care  **Population (n):** Patients who failed to attend two or more routine appointments in preceding year(173)  **Country**: Scotland, UK  **Study design:** Randomised controlled trial | **Mechanism**: Reminder, non-financial  **Time/freq**: 12 hours prior | **Results:** Multilevel analysis applied to the binary outcome data on non-attendance gave an odds ratio for non-attendance in the intervention group compared with the control group of 0.63 (95% CI 0.36 to 1.1, p = 0.11).  **Author's conclusion:** failed to demonstrate significant reduction in non-attendance rates, as a result of texting appointment reminders to patients who persistently fail to attend their general practice appointments. |
| Gerson et al. (1986)[71] | A strategy to increase appointment keeping in a pediatric clinic. | **Intervention/ comparator:** Single telephone reminder/ single postcard reminder/ both / no reminder  **Hospital dept:** Outpatient  **Population (n):** Parents of children receiving therapeutic or preventative services (253)  **Country**: USA  **Study design:** Randomised controlled trial | **Mechanism**: Reminder, non-financial  **Time/freq**: 1-2 days prior | **Results:**  **Author's conclusion:** Postcard reminders were as effective as telephone reminders |
| Griffin et al. (2011)[42] | Improving endoscopy completion: Effectiveness of an interactive voice response system | **Intervention/ comparator:** Interactive response system / nurse phonecall  **Hospital dept:** Gastrointestinal  **Population (n):** Patients scheduled for colonoscopy (3610)  **Country**: USA  **Study design:** Randomised controlled trial | **Mechanism**: Reminder, non-financial  **Time/freq**: 1 week to 3 days prior | **Results:** No statistically significant differences across the 3 study arms in appointment attendance or adherence to preparation instructions.  **Author's conclusion:** An IVR system call is as effective as a nurse phone call for ensuring that patients attend appointments and are adequately prepared for endoscopy examinations. |
| Groden et al. (2021)[27] | The success of behavioral economics in improving patient retention within an intensive primary care practice | **Intervention/ comparator:** Clinical signage and appointment reminder cards  **Hospital dept:** Primary care  **Population (n):** High cost patients (1716)  **Country**: USA  **Study design:** Non-randomised experimental study | **Mechanism**: Signage, non-financial  **Time/freq**: n/a | **Results:** The visit adherence rate increased from 74.7% at baseline to 76.5% (p=.22) during t1 and 78.0% (p=.03) during t2.  **Author's conclusion:** A low-resource, clinic-based behavioral economics intervention was capable of improving patient retention within a traditionally high-cost population. |
| Gullo et al. (2018)[72] | Short message service (SMS) reminders improve treatment attendance in alcohol dependence, but are less effective for patients high in impulsivity | **Intervention/ comparator:** SMS reminder / no reminder  **Hospital dept:** Addiction  **Population (n):** Alcohol-dependent patients (193)  **Country**: Australia  **Study design:** Non-randomised experimental study | **Mechanism**: Reminder, non-financial  **Time/freq**: 1 day prior | **Results:** SMS reminders significantly increased probability of session attendance (0.90 versus 0.84, p = .02)  **Author's conclusion:** SMS appointment reminders improve treatment attendance for alcohol-dependent outpatients. |
| Hallsworth et al. (2015)[6] | Stating appointment costs in SMS reminders reduces missed hospital appointments: Findings from two randomised controlled trials | **Intervention/ comparator:** Three different SMS reminders easy call, social norm and specific cost / no message  **Hospital dept:** Outpatient  **Population (n):** outpatients with valid mobile phone number (10,111)  **Country**: UK  **Study design:** Randomised controlled trial | **Mechanism**: Reminder, non-financial  **Time/freq**: n/a | **Results:** a message including the cost of a missed appointment to the health system produced a DNA rate of 8.4%, compared to 11.1% for the existing message (OR 0.74, 95%CI 0.61–0.89, P<0.01).  **Author's conclusion:** Missed appointments can be reduced, for no additional cost, by introducing persuasive messages to appointment reminders. |
| Hashim et al. (2001)[33] | Effectiveness of telephone reminders in improving rate of appointments kept at an outpatient clinic: a randomized controlled trial. | **Intervention/ comparator:** Telephone reminder/ no telephone reminder  **Hospital dept:** Primary care  **Population (n):** All patients with appointments (903)  **Country**: USA  **Study design:** Randomised controlled trial | **Mechanism**: Reminder, non-financial  **Time/freq**: 1 day prior | **Results:** No show was 19% in the telephoned and 26% in the not-telephoned groups (P5.0065). Significantly more cancelations were made when telephoning patients before their visit, 17% compared with 9.9%.  **Author's conclusion:** Reminding patients by telephone calls 1 day before their appointments yields increased cancelations that can be used to schedule other patients. |
| Horvath et al. (2011)[73] | Impact of health portal enrollment with email reminders on adherence to clinic appointments: a pilot study. | **Intervention/ comparator:** Health View portal users / no health view portal users  **Hospital dept:** Outpatient  **Population (n):** all patients attending one of seven Duke Medicine clincis (58,942)  **Country**: USA  **Study design:** Cross sectional study | **Mechanism**: Agency, non-financial  **Time/freq**: n/a | **Results:** The adjusted odds of arrival increased 39.0%for portal enrollees relative to nonusers (OR = 1.39, 95% CI 1.22 - 1.57, P< .001).  **Author's conclusion:** In combination with an email reminder feature, online portal use may have an important and beneficial effect on clinic operations |
| Junod Perron et al. (2013) [17] | Text-messaging versus telephone reminders to reduce missed appointments in an academic primary care clinic: a randomized controlled trial. | **Intervention/ comparator:** SMS reminder/ no reminder  **Hospital dept:** Primary care  **Population (n):** Patients registered for appointments (6450)  **Country**: Switzerland  **Study design:** Randomised controlled trial | **Mechanism**: Reminder, non-financial  **Time/freq**: 1 day prior | **Results:**The rate of missed appointments was similar in the text-message group (11.7%, 95% CI: 10.6-12.8) and in the telephone group (10.2%, 95% CI: 9.2-11.3 p = 0.07).  **Author's conclusion:** Text-message reminders are equivalent to telephone reminders in reducing the proportion of missed appointments in an academic primary care clinic and are more cost-effective. |
| Koren et al. (1994)[37] | Interventions to improve patient appointments in an ambulatory care facility | **Intervention/ comparator:** Phone call reminder/ postcard reminder/ no reminder  **Hospital dept:** Outpatient  **Population (n):** Patients seen by medical subspeciality outpatient physicians (956)  **Country**: USA  **Study design:** Randomised controlled trial | **Mechanism**: Reminder, non-financial  **Time/freq**: 2-3 days prior (phone), 5 days prior postcards | **Results:** 74.1% of control group kept scheduled appointment vs 82.1% in phonecall group or 81.1% in postcard group  **Author's conclusion:** Patient reminders in the form of a phone call or postcard have been shown to have a significant impact on the no-show rate in a medical ambulatory care center. |
| Kourany et al. (1990)[40] | Improving first appointment attendance rates in child psychiatry outpatient clinics | **Intervention/ comparator:** Telephone reminder/ letter reminder/ phone call and letter/ no contact  **Hospital dept:** Mental health  **Population (n):** Consecutive patients calling to make appointments (111)  **Country**: USA  **Study design:** Randomised controlled trial | **Mechanism**: Reminder, non-financial  **Time/freq**: 1-2 days prior | **Results:** the effect of any kind of contact (call, letter or both) versus no contact was significant, x2(2)=7.05. p <0.03.  **Author's conclusion:** Any contact was significantly more likely to reduce the "no show" rate than no contact. |
| Kravariti et al. (2018)[76] | Effectiveness of automated appointment reminders in psychosis community services: A randomised controlled trial | **Intervention/ comparator:** Automated text message reminder/ no reminder  **Hospital dept:** Mental health  **Population (n):** Patients with mobile phones, willing to receive SMS (95)  **Country**: UK  **Study design:** Randomised controlled trial | **Mechanism**: Reminder, non-financial  **Time/freq**: 1 week prior, again 1 day prior | **Results:** OR remained robust and statistically significant (adjusted OR = 2.95, 95% CI 1.05–8.85,P<0.05).  **Author's conclusion:** Automated messaging reminders provide a feasible and robust strategy for engaging people with psychosis in healthcare. |
| Krishna & Amarjothi (2012)[31] | Reminder letters to imprve rate of attendance at community mental health centre | **Intervention/ comparator:** Reminder letter/ no reminder letter  **Hospital dept:** Mental health  **Population (n):** Adults attending mental health center (1466)  **Country**: UK  **Study design:** Non-randomised experimental study | **Mechanism**: Reminder, non-financial  **Time/freq**: 1 week prior | **Results:** The attendance rate in the experimental group was 71.95% (585/813) as opposed to 56.57% (344/555) in the control group (OR=1.57; p=0.0001).  **Author's conclusion:** Sending reminder letters within a week of the next appointment to patients improved attendance significantly |
| Kwon et al. (2012)[18] | The effect of telephone call reminders on electrodiagnostic laboratory attendance in Korea | **Intervention/ comparator:** Single telephone call reminder/ no reminder  **Hospital dept:** Neurological  **Population (n):** All patients scheduled for electrodiagnostic exams (404)  **Country**: Korea  **Study design:** Randomised controlled trial | **Mechanism**: Reminder, non-financial  **Time/freq**: 1 day prior | **Results:** The attendance rate was found to be significantly higher for the needle EMG reminded group (97.6% (42/43) versus 75.9% (22/29)) (OR 0.07; 95% CI 0.01, 0.61; P = 0.016).  **Author's conclusion:** Although the effect of telephone reminders was not found to be significant in terms of overall appointment attendances at an EDX laboratory, telephone reminders greatly improved attendance for needle EMG examinations. |
| Lam et al (2021)[78] | Short Message Service reminders reduce outpatient colonoscopy nonattendance rate: A randomized controlled study | **Intervention/ comparator:** SMS reminder/ no reminder  **Hospital dept:** Colonoscopy  **Population (n):** Patients offered outpatient colonoscopy appointment (2225)  **Country**: Hong Kong, China  **Study design:** Randomised controlled trial | **Mechanism**: Reminder, non-financial  **Time/freq**: 1-1.5 weeks prior | **Results:** The nonattendance rate of patients in the SMS group was significantly lower than in the standard care group.  **Author's conclusion:** An SMS reminder for outpatient colonoscopy is effective in reducing the nonattendance rate and may potentially improve the bowel preparation quality. |
| Lance et al. (2021)[38] | Comparison Between Short Text Messages and Phone Calls to Reduce No-Show Rates in Outpatient Medical Appointments A Randomized Trial | **Intervention/ comparator:** Telephone or SMS reminder/ no reminder  **Hospital dept:** Primary care  **Population (n):** Adult patients with internal medicine appointments (306**)**  **Country**: Brazil  **Study design:** Randomised controlled trial | **Mechanism**: Reminder, non-financial  **Time/freq**: 2 days prior | **Results:** The lowest percentage of no-show (9.5%) occurred in the telephone call group, while the SMS group presented at 21% and the no-intervention group at 22.8% (P=.025)  **Author's conclusion:** The use of tele-phone calls has proved to be more efficient and effective than SMS |
| Lee et al. (2020)[25] | A financial incentive program to improve appointment attendance at a safety-net hospital-based primary care hepatitis C treatment program | **Intervention/ comparator:** $15 gift card to attending appointment / no financial reward  **Hospital dept:** Primary care  **Population (n):** Patients receiving hepatitis c treatment with active infections (327)  **Country**: USA  **Study design:** Non-randomised experimental study | **Mechanism**: Incentive, Financial (reward)  **Time/freq**: n/a | **Results:** Appointments in the intervention group were more likely to be attended.  **Author's conclusion:** Implementation of a financial incentive program was associated with improved appointment attendance at a safety-net hospital-based primary care HCV treatment program. |
| Leong et al (2006)[35] | The use of text messaging to improve attendance in primary care: a randomized controlled trial. | **Intervention/ comparator:** SMS reminders / no reminders  **Hospital dept:** Primary care  **Population (n):** Patients requiring follow up appointments (993)  **Country**: Malaysia  **Study design:** Randomised controlled trial | **Mechanism**: Reminder, non-financial  **Time/freq**: 1-2 days prior | **Results:** Attendance rate of the text messaging reminder group was significantly higher compared with that of the control group (odds ratio 1.59, 95% confidence interval 1.17 to 2.17,P= 0.005).There was no statistically significant difference in attendance rates between text messaging and mobile phone reminder groups.  **Author's conclusion:** Text messaging reminder system was effective in improving attendance rate in primary care. It was more cost-effective compared with the mobile phone reminder. |
| Liew et al. (2009)[39] | Text messaging reminders to reduce non-attendance in chronic disease follow-up: A clinical trial | **Intervention/ comparator:** Telephone reminder / text message reminder  **Hospital dept:** Primary care  **Population (n):** registered clinic patients with chronic disease (931)  **Country**: Malaysia  **Study design:** Randomised controlled trial | **Mechanism**: Reminder, non-financial  **Time/freq**: 1-2 days prior | **Results:** The non-attendance rates in the text messaging group (odds ratio[OR]=0.62,95% confidence interval[CI]=0.41 to 0.93,P=0.020) and the telephone reminder group(OR=0.53,95%CI=0.35 to 0.81),P=0.003) were significantly lower than the control group  **Author's conclusion:** Text messaging was found to be as effective as telephone reminder in reducing non-attendance in patients who required long-term follow-up for their chronic illnesses in this study. |
| Mahmud et al. (2021)[22] | Effect of Text Messaging on Bowel Preparation and Appointment Attendance for Outpatient Colonoscopy: A Randomized Clinical Trial | **Intervention/ comparator**: Additional automated education and reminder text messages/ usual care  **Hospital dept:** Gastrointestinal  **Population (n):** All patients schedule for outpatient colonoscopy (753)  **Country**: USA  **Study design:** Randomised controlled trial | **Mechanism**: Reminder, non-financial  **Time/freq**: 1 week prior | **Results:** patients attending appointments with good or excellent bowel preparation: intervention, 195 patients [53.1%]; control, 210 patients [54.4%];P= .73)  **Author's conclusion:** Found no significant difference in appointment attendance or bowel preparation quality with an automated text messaging intervention compared with the usual care control. |
| Mikhaeil et al. (2019)[32] | Attend: A Two-Pronged Trial to Eliminate No Shows in Diagnostic Imaging at a Community-Based Hospital | **Intervention/ comparator:** Pre and post implementation of mailed reminder and patient information pamphlet  **Hospital dept:** Diagnostic imaging  **Population (n):** Patients attending diagnostic imaging appointments (not reported)  **Country**: Canada  **Study design:** Non-randomised experimental study | **Mechanism**: Reminder, non-financial  **Time/freq**: n/a | **Results:** The mailing letter resulted in a significant reduction from 7.1% to 6.3% in overall no shows across two community hospitals(P=.04).  **Author's conclusion:** Both the methods, the mailing letter and patient-information pamphlet, provide promising results in regard to reducing the no-show percentage among patients |
| Milne (2010)[80] | Reducing non-attendance at specialist clinics: an evaluation of the effectiveness and cost of patient-focused booking and SMS reminders at the Scottish health board | **Intervention/ comparator:** Last minute SMS and email reminders (patient focused booking)/ no reminders  **Hospital dept:** Outpatient services  **Population (n):** (73979)  **Country**: Scotland, UK  **Study design:** Non-randomised experimental study | **Mechanism**: Reminder, non-financial  **Time/freq**:n/a | **Results:** Reduced probability of non-attendance to 0.258 of what it would have been under status quo.  **Author's conclusion:** SMS reminders and booking appointments no more than 6 weeks in advance can be effective and low-cost way to reduce non-attendance. |
| Narring et al. (2013)[19] | Text-messaging to reduce missed appointment in a youth clinic: A randomised controlled trial | **Intervention/ comparator:** SMS reminder/ no reminder  **Hospital dept:** Youth clinic  **Population (n):** Patients between 12-24 years (999)  **Country**: Switzerland  **Study design:** Randomised controlled trial | **Mechanism**: Reminder, non-financial  **Time/freq**: 1 day prior | **Results:** The proportion of missed appointments was16.4% (95% CI 13.1% to 19.8%) in the text-message group (N 462) and 20.0% (95% CI 16.6% to 23.4%)in the control group (N 529), showing no significant effect of the intervention (p=0.346).  **Author's conclusion:** In a primary care youth clinic, text-message reminders are not effective in reducing the proportion of missed appointments. |
| Nayor et al. (2019)[81] | Impact of Automated Time-released Reminders on Patient Preparedness for Colonoscopy | **Intervention/ comparator:** Automated time released reminder text message and email/ no electornic reminders  **Hospital dept:** Gastrointestinal  **Population (n):** All patients scheduled for colonoscopy(1497)    **Country**: USA  **Study design:** Non-randomised experimental study | **Mechanism**: Reminder, non-financial  **Time/freq**: 2 weeks to one day prior | **Results:** The automated reminder program improved adequate preparation quality 2.85-fold (95% confidence interval, 2.03-3.99;P<0.0001)  **Author's conclusion:** Implementation of automated time-released colonoscopy preparation reminders via text messages and emails improved patient preparedness for colonoscopy, with significantly improved bowel preparation quality and fewer canceled procedures |
| Nelson et al. (2011)[36] | Assessing the effectiveness of text messages as appointment reminders in a pediatric dental setting | **Intervention/ comparator:** Text message reminder/ voice reminder  **Hospital dept:** Dentistry  **Population (n):** Caregivers of patients at paediatric dental clinic (543)  **Country**: USA  **Study design:** Case control study | **Mechanism**: Reminder, non-financial  **Time/freq**: 2 days prior | **Results:** Participants who received text message reminders were 2.12 times as likely not to attend their dental appointment  **Author's conclusion:** SMS text messages were not as effective as voice reminders for patients in a dental school pediatric dentistry clinic |
| Parikh et al. (2010)[43] | The Effectiveness of Outpatient Appointment Reminder Systems in Reducing No-Show Rates | **Intervention/ comparator:** Reminders by telephone/ auto reminder  **Hospital dept:** Outpatient  **Population (n):** Patients schedule with outpatient appointments (12,092)  **Country**: USA  **Study design:** Randomised controlled trial | **Mechanism**: Reminder, non-financial  **Time/freq**: 3 days prior | **Results:** The no-show rates for patients in the STAFF, AUTO, and NONE groups were 13.6%, 17.3%, and 23.1%, respectively.  **Author's conclusion:** A clinic staff reminder was significantly more effective in lowering the no-show rate compared with an automated appointment reminder system |
| Percac-Lima et al. (2015)[82] | Patient navigation based on predictive modeling decreases no-show rates in cancer care | **Intervention/ comparator:** Two reminder calls from navigators/ standard of care  **Hospital dept:** Outpatient  **Population (n):** Patients likely to miss schedule appointment (4425)  **Country**: USA  **Study design:** Randomised controlled trial | **Mechanism**: Reminder, non-financial  **Time/freq**: 1 week and again 1 day prior | **Results: T**he no-show rate in the intervention group was 10.2% (167 of 1631), compared with 17.5% in the control group (280 of 1603) (P<.001).  **Author's conclusion:** Telephone navigation targeted at those patients predicted to be at high risk of visit nonadherence was found to effectively and substantially improve patient adherence to cancer clinic appointments. |
| Percac-Lima et al. (2016)[83] | Can Text Messages Improve Attendance to Primary Care Appointments in Underserved Populations? | **Intervention/ comparator**: additional text message reminders/ phone call reminder only  **Hospital dept:** Primary care  **Population (n):** All adult medicine schedule appointments (11104)  **Country**: USA  **Study design:** Randomised controlled trial | **Mechanism**: Reminder, non-financial  **Time/freq**:1 week and again 1 day prior | **Results:** The no- show rate in the matched control group was 20.2% (265 / 1309) compared with 13.8% (180 / 1309) for those in the intervention group that accepted text message (difference in proportions = 6.4%, CI 3.63% to 9.36%, p = .001).  **Author's conclusion:** Text message reminders significantly improved attendance at primary care appointments in patients who agreed to receive. |
| Perron et al. (2010)[45] | Reduction of missed appointments at an urban primary care clinic: a randomised controlled study. | **Intervention/ comparator:** Phone call, SMS and postal reminder/ no reminder  **Hospital dept:** Primary care/ HIV  **Population (n):** All patients schedule for appointments (2130)  **Country**: Switzerland  **Study design:** Randomised controlled trial | **Mechanism**: Reminder, non-financial  **Time/freq**: 2 days prior | **Results:** intervention significantly reduced the rate of missed appointments from 122/1071 (11.4%) to 82/1052(7.8%; p < 0.005).  **Author's conclusion:** |
| Quattlebaum et al. (1991)[28] | Effectiveness of computer-generated appointment reminders | **Intervention/ comparator:** Mailed computer generated appointment reminder/ no reminder  **Hospital dept:** Primary care  **Population (n):** General paediatric patients (432)  **Country**: USA  **Study design:** Non-randomised experimental study | **Mechanism**: Reminder, non-financial  **Time/freq**: 3 days prior | **Results:** No-show rate was reduced from 19% in the control group to 10% in the reminder group, representing a 48% reduction (p =.0002**)**  **Author's conclusion:** Computer-generated appointment reminders were found to lead to large reductions in no-show rates among all categories of patients. |
| Ritchie et al. (2000)[86] | A telephone call reminder to improve outpatient attendance in patients referred from the emergency department: A randomised controlled trial | **Intervention/ comparator:** Telephone call reminder / usual care  **Hospital dept:** Outpatient  **Population (n):** Patients advised by ED doctors to make outpatient appointment (400)  **Country**: Australia  **Study design:** Randomised controlled trial | **Mechanism**: Reminder, non-financial  **Time/freq**: 1-3 days prior | **Results:** Improved attendance at scheduled appointments from 54.4% to 70.7% (p0.002).  **Author's conclusion:** A significant improvement in the proportion of patients attending outpatients appointments can be made by a simple reminder telephone call one to three days after attendance at the ED. |
| Roberts et al. (2007)[62] | The effect of telephone reminders on attendance in respiratory outpatient clinics | **Intervention/ comparator:** Telephone call reminder / usual care  **Hospital dept:** Respiratory  **Population (n):** All new and follow up patients from four clinics (504)  **Country**: UK  **Study design:** Randomised controlled trial | **Mechanism**: Reminder, non-financial  **Time/freq**: 1 week prior | **Results:** There was a significant 15% increase in attendance in the contacted group (n = 104) when compared both with the control group (71%, n = 258)  **Author's conclusion:** Routine telephoning of outpatients should become standard practice if reducing non-attendance is thought to be desirable |
| Roseland et al. (2022) [24] | Targeting Missed Care Opportunities Using Modern Communication Methods: A Quality Improvement Initiative to Improve Access to CT and MRI Appointments | **Intervention/ comparator:** Automated message, phonecall and text message/ human initiated calls  **Hospital dept:** Radiology/ Diagnostics  **Population (n):** Adult outpatients schedule for CT or MRI exam (21067**)**  **Country**: USA  **Study design:** Non-randomised experimental study | **Mechanism**: Reminder, non-financial  **Time/freq**: 1 week prior | **Results:** Automated messaging did not significantly change the proportion of missed care opportunities for CT (traditional: 2.62% [95% CI: 2.233.06] vs. automated: 2.06%[95% CI: 1.702.48],p= 0.05) or MRI (traditional: 3.1% [95% CI: 2.603.66] vs. automated: 2.83% [95% CI: 2.403.30],p= 0.43).  **Author's conclusion:** Automated messaging did not meaningfully change the overall proportion of missed care opportunities compared to traditional human-initiated phone calls, however they may reduce cost and improve efficiency without adversely affecting access to care |
| Ruggeri et al. (2020)[34] | Nudging New York: adaptive models and the limits of behavioral interventions to reduce no-shows and health inequalities | **Intervention/ comparator:** Before and after robocall reminder intervention  **Hospital dept:** Primary care  **Population (n):** Patients at federally qualified health centers (3842)  **Country**: USA  **Study design:** Retrospective observational study | **Mechanism**: Reminder, non-financial  **Time/freq**: 3 days and again 2 days prior | **Results:** There was no significant effect of the reminder on no-show rates. The no-show rate for appointments prior to the reminder was 41.6, and 42.1% after the reminder was implemented  **Author's conclusion:** The limited effects of the reminder intervention suggest the need for more personalized behavioral interventions to reduce no-shows. |
| Rusius (1995)[29] | Improving out-patient attendance using postal appointment reminders | **Intervention/ comparator:** Postal reminder 3 days prior/ standard appointment letter within 2 weeks  **Hospital dept:** Mental health  **Population (n):** Adult psychiatric outpatient (144)  **Country**: UK  **Study design:** Randomised controlled trial | **Mechanism**: Reminder, non-financial  **Time/freq**: 3 days prior | **Results:** Difference in the rates of attendance and non-attendance in the two groups is statistically significant at 0.05>P>0.02  **Author's conclusion:** Sending of appointment reminders may be a cost-effective way of increasing attendance and reducing non-attendance for new referrals to a psychiatric out-patient clinic. |
| Senderey et al. (2020)[46] | It's how you say it: Systematic A/B testing of digital messaging cut hospital no-show rates | **Intervention/ comparator:** Eleven different framings of SMS reminders  **Hospital dept:** Primary care  **Population (n):** Adult patients with outpatient appointments (2247)  **Country**: Israel | **Mechanism**: Reminder, non-financial  **Time/freq**: n/a | **Results:** Members who received a reminder designed to evoke emotional guilt had a no-show rates of 14.2%, compared with 21.1% in the control group (odds ratio [OR]: 0.69, 95% confidence interval [CI]: 0.67, 0.76), and an advanced cancellation rate of 26.3% compared with 17.2% in the control group (OR: 1.2, 95% CI: 1.19, 1.21).  **Author's conclusion:** Careful design of SMS narratives based on behavioral economic principles can reduce hospital outpatient clinic no-show rates by over 30 percent. |
| Shah et al. (2016)[88] | Targeted Reminder Phone Calls to Patients at High Risk of No-Show for Primary Care Appointment: A Randomized Trial | **Intervention/ comparator:** Clinical staff call/ automated phone call  **Hospital dept:** Primary care  **Population (n):** Adult primary care patients with high risk of no-show (2247)  **Country**: USA  **Study design:** Randomised controlled trial | **Mechanism**: Reminder, non-financial  **Time/freq**: 1 week prior | **Results:** The no-show rate in the intervention arm(22.8 %) was significantly lower (absolute risk difference−6.4 %,p< 0.01, 95 % CI [−9.8 to−3.0 %]) than that in the control arm (29.2 %).  **Author's conclusion:** A phone call 7 days prior to an appointment led to a significant reduction in no-shows and increased reimbursement among patients at high risk of no-show. |
| Sims et al. (2012)[61] | Text message reminders of appointments: A pilot intervention at four community mental health clinics in London | **Intervention/ comparator:** 4 months pre SMS reminder intervention/ same 4 months 1 year later.  **Hospital dept:** Mental health  **Population (n):** Outpatient appointments at four clinics (2817)  **Country**: UK  **Study design:** Non-randomised experimental study | **Mechanism**: Reminder, non-financial  **Time/freq**: 1 week and again 5 days prior | **Results:** The relative risk reduction in failed attendance was 28% between the 2008 and 2009 samples and25% between the 2008 and 2010 samples.  **Author's conclusion:** SMS-based technology can offer a time-, labor-, and cost-efficient strategy for encouraging engagement with psychiatric outpatient services. |
| Steiner et al. (2016)[89] | Reducing missed primary care appointments in a learning health system | **Intervention/ comparator:** Single interactive voice response (IVR) technology call reminder / no reminder  **Hospital dept:** Primary care  **Population (n):** Adults with primary care appointments (8804)  **Country**: USA  **Study design:** Randomised controlled trial | **Mechanism**: Reminder, non-financial  **Time/freq**: 1 day prior | **Results:** Patients receiving IVR-T had a lower rate of missed appointments than those receiving no reminder at the IC (6.5% vs.7.5%, relative risk = 0.85, 95% confidence interval, 0.72–1.00) and RC (8.2% vs. 10.5%, relative risk = 0.76, 95% confidence interval,0.65–0.89).  **Author's conclusion:** A single IVR-T call reduced missed appointments |
| Steiner et al. (2018)[90] | Optimizing number and timing of appointment reminders: A randomized trial | **Intervention/ comparator:** Interactive voice response or text message 3 days prior vs 1 day prior vs 2 reminders-- 3 days and 1 day before  **Hospital dept:** Primary care  **Population (n):** All patients with primary care appointments (54066)  **Country**: USA  **Study design:** Randomised controlled trial | **Mechanism**: Reminder, non-financial  **Time/freq**: 1-3 days prior | **Results:** 2 reminders, delivered by text message or telephone 3 days and 1 day prior to a primary care visit, were more effective than a single reminder delivered either 3 days or 1 day prior to a visit.  **Author's conclusion:** Text messages and telephone calls were equally effective in reducing the rate of missed appointments |
| Stormon et al.(2021)[23] | SMS reminders to improve outpatient attendance for public dental services: A retrospective study | **Intervention/ comparator:** 12 months before and after intervention of SMS reminder asking to confirm appointment  **Hospital dept:** Dentistry  **Population (n):** Adolescent and adult services patients (9287)  **Country**: Australia  **Study design:** Non-randomised experimental study | **Mechanism**: Reminder, non-financial  **Time/freq**: 1 day prior | **Results:** The rate of attendance during the post-SMS reminder period was lower (72.6 per 100 attendances) than the 75.7 per 100 during the pre SMS period.  **Author's conclusion:** SMS reminders had a mixed effect on children and adult's attendance rates. |
| Tan et al.(2019)[91] | Automated Text Message Reminders Improve Radiation Therapy Compliance | **Intervention/ comparator:** Automated SMS message delivered daily / no reminder  **Hospital dept:** Radiation  **Population (n):** Patients treated at UT Southwestern medical center (3400)  **Country**: USA  **Study design:** Non-randomised experimental study | **Mechanism**: Reminder, non-financial  **Time/freq**: 2 hours prior | **Results:** Nonreceipt of SMS appointment reminders had a strong association no-shows (OR, 6.77; 95% CI, 5.45-8.41;P<.0001)  **Author's conclusion:** Receipt of text messages correlates with compliance for radiation therapy appointments. |
| Taylor et al. (2012)[92] | Mobile telephone short message service reminders can reduce nonattendance in physical therapy outpatient clinics: A randomized controlled trial | **Intervention/ comparator:** SMS reminder message / no reminder message  **Hospital dept:** Physical therapy  **Population (n):** Patients with physical therapy appointment (679)  **Country**: Australia  **Study design:** Randomised controlled trial | **Mechanism**: Reminder, non-financial  **Time/freq**: 2 days prior | **Results:** Patients not receiving a reminder were 1.61 times more likely to not attend their next appointment without canceling than patients who received the SMS reminder (OR, 1.61; 95%CI, 1.03-2.51).  **Author's conclusion:** SMS reminders can reduce nonattendance in physical therapy outpatient clinics |
| Teeng et al. (2021)[93] | Reminder through mobile messaging application improves outpatient attendance and medication adherence among patients with depression: An open-label randomised controlled trial | **Intervention/ comparator:** Mobile messaging reminder / no reminder  **Hospital dept:** Mental health  **Population (n):** Adult patients diagnosed with major depressive disorder (183)  **Country**: Malaysia  **Study design:** Randomised controlled trial | **Mechanism**: Reminder, non-financial  **Time/freq**: 1 day prior | **Results:** At two months, the increase in outpatient attendance rate of intervention group was 20% higher than that of control group(p=0.002).  **Author's conclusion:** Reminders through mobile messaging applications improved both outpatient attendance and medication adherence among patients with depression, resulting in greater improvement of depressive symptoms |
| Teo et al. (2017)[94] | No-show rates when phone appointment reminders are not directly delivered | **Intervention/ comparator:** Live reminder, message reminder, no answer  **Hospital dept:** Mental health, Veterans Health Administration  **Population (n):** Patients with depression (250)  **Country**: USA  **Study design:** Non-randomised experimental study | **Mechanism**: Reminder, non-financial  **Time/freq**: 1 day prior | **Results:** Live reminders were associated with the lowest no-show rate (3%), whereas no-show rates were significantly higher for message reminders (24%) and no answer (39%).  **Author's conclusion:** Appointment attendance rates were considerably higher when there was a live contact |
| Thomas et al. (2017)[95] | Effect of short message service reminders on clinic attendance among outpatients with psychosis at a psychiatric hospital in Nigeria | **Intervention/ comparator:**  SMS reminders about an upcoming appointment vs current standard procedure of appointment cards containing the appointment date written by hand  **Hospital dept:** Mental health  **Population (n):** Adults seeking treatment for psychotic illness (192)  **Country**: Nigeria  **Study design:** Randomised controlled trial | **Mechanism**: Reminder, non-financial  **Time/freq**: 5 and 3 days prior | **Results:** The odds of attending the appointment were significantly greater among participants who received an SMS reminder compared with the control group (odds ratio [OR]=1.80, CI=1.02–3.19)  **Author's conclusion:** SMS reminders of clinic appointment dates could be an effective strategy in Nigeria to encourage patients with FEP to attend clinic appointments as scheduled. |
| Youssef (2014)[97] | Use of short message service reminders to improve attendance at an internal medicine outpatient clinic in Saudi Arabia: A randomized controlled trial | **Intervention/ comparator:** SMS reminder message / no reminder message  **Hospital dept:** Outpatient  **Population (n):** Arabic speaking adult patients (502)  **Country**: Saudi Arabia  **Study design:** Randomised controlled trial | **Mechanism**: Reminder, non-financial  **Time/freq**: 2 days prior | **Results:** Multivariate logistic regression analysis showed that patients who received SMS reminders were 44% less likely to miss their appointment compared with those who did not receive SMS reminder (OR = 0.56, 95% CI: 0.38–0.82, P = 0.001)  **Author's conclusion:** SMS reminders were effective in reducing the non-attendance rate at internal medicine outpatient clinic appointments in a major Saudi hospital |
| Youssef et al. (2014)[98] | Effectiveness of text message reminders on nonattendance of outpatient clinic appointments in three different specialties: A randomized controlled trial in a Saudi Hospital | **Intervention/ comparator:** SMS reminder message / no reminder message  **Hospital dept:** Outpatient  **Population (n):** Patients due at outpatient clinics (1499)  **Country**: Saudi Arabia  **Study design:** Randomised controlled trial | **Mechanism**: Reminder, non-financial  **Time/freq**: 2 days prior | **Results:** GM clinic (nonattendance of 39.8% vs. 26.3% for control and intervention groups respectively, P< 0.001) and in Neurology clinic (nonattendance of 43.9% vs. 29.3% for the control and intervention groups **r**espectively, P= 0.02  **Author's conclusion:** The use of SMS reminders for outpatient appointments was associated with a significant reduction in nonattendance rate, although it varies by clinic specialty. |
